# An Update on Diabetic Women Obstetrical Outcomes Linked to Preconception and Pregnancy Glycemic Profile: A Systematic Literature Review

**DOI:** 10.1155/2013/254901

**Published:** 2013-11-06

**Authors:** Salvatore Gizzo, Tito Silvio Patrelli, Marta Rossanese, Marco Noventa, Roberto Berretta, Stefania Di Gangi, Martina Bertin, Michele Gangemi, Giovanni Battista Nardelli

**Affiliations:** ^1^Department of Woman and Child Health, University of Padua, Via Giustiniani 3, 35128 Padua, Italy; ^2^Department of Surgical Sciences, University of Parma, Parma, Italy; ^3^Dipartimento di Scienze Chirurgiche, U.O.C. di Ginecologia e Ostetricia, Via Gramsci 14, 43100 Parma, Italy

## Abstract

Women with type 2 diabetes were less likely to have diabetes related complications than women with type 1. Women with type 1 diabetes had a high prepregnancy care and showed a worse glycemic control than women with type 2 both in the preconception period and during pregnancy. Obstetrical outcomes showed that preeclampsia and stillbirth rate is almost doubled in type 1 patients while perinatal deaths and SGA importantly increased in type 2 diabetes. In modern obstetrical care it is mandatory to maintain glucose levels as close to normal as possible particularly in diabetic population. HbA1C no higher than 6% before pregnancy and during the first trimester seems to decrease the risk of adverse obstetrical outcomes. Both the preconceptional counseling and glycemic profile optimization represent a fundamental step to improve pregnancy outcomes in women with preexisting diabetes. A systematic approach to family planning and the availability of preconception care for all diabetic women who desire pregnancy could be an essential step for diabetic management program.

## 1. Introduction

Diabetes is a metabolic disease determined by defects in insulin secretion, insulin action, or both, which caused a chronic hyperglycemia with a long-term damage, dysfunction, and failure of different organs. The American Diabetes Association had published a classification of diabetes, which considered four major classes: type 1 diabetes, type 2 diabetes, gestational diabetes mellitus, and other specific types of diabetes [1].

According to the International Diabetes Federation (IDF) the worldwide prevalence of diabetes mellitus in 2011 resulted in 366 millionof cases and projections speculate that in 2030 the prevalence of this metabolic disease will reach 552 million of cases (estimated prevalence of about 7.7%) [2]. According to this evidence, in USA population in the years 1999–2005, the pregnant affected by diabetes ranged from 10% to 21%, suggesting that in the next future the prevalence of diabetic women who became pregnant will be increased [3].

Pregnancy is physiologically characterized by increased insulin resistance and reduced sensitivity to insulin action, due to the effects of placental hormones, like human placental lactogen, progesterone, prolactin, placental growth hormone, and cortisol. This change in maternal metabolism is directed towards supplying adequate nutrition for the fetus [4]. 

Much evidence reported that pregnancies in women with preexisting diabetes, both type 1 and type 2, are affected by an increased risk of maternal and fetal adverse outcomes, probably linked to poor glycemic control, especially in periconceptional period and in the first trimester of pregnancy [5]. 

The most common maternal complications reported were preeclampsia, spontaneous preterm labor, operative delivery, and Cesarean delivery (CD), while fetal and neonatal frequent complications resulted in miscarriages, congenital anomalies, macrosomia, small for gestational age (SGA), and stillbirth [6]. 

Furthermore, women with preexisting diabetes could have an exacerbation of many diabetes-related complications such as retinopathy, nephropathy, or chronic hypertension [6]. 

The first goal of this review is to evaluate both maternal demographic characteristics and glycemic control in a preconception diabetic cohort of pregnant in order to identify a possible correlation with the obstetric outcomes. 

As second intent, we evaluated the glucose levels (both preconception and during pregnancy) in order to detect the most appropriate cut-off that should be targeted to improve pregnancy outcomes.

## 2. Data Sources

We performed a systematic research in the electronic databases MEDLINE, EMBASE, ScienceDirect, and the Cochrane Library concerning pregnancy outcomes in women with pregestational diabetes (types 1 and 2) in interval time between 2000 and 2012. 

Key search terms included pregestational diabetes, pregnancy outcome, glycosylated hemoglobin serum value (HbA1c), miscarriages, Cesarean delivery, preterm delivery, malformations, macrosomia, small for gestational age, stillbirths, and perinatal deaths.

A manual search of reference lists of included studies and review articles was also performed. References from the retrieved articles were searched to identify any articles excluded by the initial search. The electronic search was performed by one of the authors blinded to aim of the study while the detection of eligible studies was performed by another one.

### 2.1. Inclusion and Exclusion Criteria

We included studies that fulfilled the following criteria: studies providing data about pregnancies in women with type 1 or type 2 diabetes mellitus, with an arbitrary minimum number of 45 patients enrolled for each study. All eligible studies had to report HbA1c serum value or fasting glucose level and the rate of women with systemic disease such as retinopathy, nephropathy, and hypertension. 

We excluded studies reporting data about gestational diabetes.

### 2.2. Outcomes of Interest and Data Collection

We looked for studies providing data about the following outcomes: miscarriages, CD, preterm delivery, malformations, macrosomia, SGA, stillbirths, and perinatal deaths. We considered data about both demographic characteristics of pregnant women (age, body mass index (BMI), years of diabetes, preconception care, planned pregnancy, nulliparous, duration of gestation, comorbidities) and metabolic control of them (HbA1c serum value before pregnancy, and during periconception period, during first, second and third trimester of pregnancy).

Studies providing ambiguous or insufficient data about considered outcomes were excluded.

## 3. Results

Our search retrieved 344 studies. Of these, after accurate evaluation, only 14 studies were eligible for the review according to our inclusion and exclusion criteria [7–20].

All considered studies were conducted in Europe, United States, and Africa but study population was heterogeneous for ethnicity.

On basis of study design, we detected 9 prospective observational/randomized studies [7–11, 14, 15, 19, 20] and 5 retrospective cohort studies [12, 13, 16–18]. Three studies [7, 10, 11] reported data about only women with type 1 diabetes, eight studies [8, 12–14, 17–20] reported data about women with both type 1 and type 2 diabetes (considered separately), and three studies [9, 15, 16] reported data about women with both type 1 and type 2 diabetes (considered together). 

We analyzed data about 4865 women with type 1 diabetes and 1244 women with type 2. Only in 201 patients is the kind of diabetes reported.

### 3.1. Maternal Characteristics by Type of Diabetes

Considering the maternal demographic characteristics, age was reported in 12 studies, BMI in 8, duration of diabetes in 11, rate of prepregnancy care in 8, rate of planned pregnancy in 3, parity in 6, gestational age in 5, and retinopathy/nephropathy in 8.

A comparison of data showed that women with type 2 diabetes were older and heavier, had a shorter duration of diabetes, and were more frequently multiparous with respect to women with type 1. 

Women with type 2 diabetes were less likely to have diabetes-related complications than women with type 1 (21.8% versus 5.78% of retinopathy and 6.47% versus 5.04% of nephropathy).

More frequently women with type 1 diabetes had high rate of prepregnancy care (38.2% versus 19.8%); anyway there was a small difference between their reproductive programs (69.7% planned pregnancy in type 1 versus 50.9% planned pregnancy in type 2). 

Detailed data about maternal characteristics were reported in Table 1.

### 3.2. Metabolic Control by Type of Diabetes

Concerning metabolic control, 3 studies [11, 16, 20] reported serum levels of HbA1c before pregnancy, 4 studies [9, 13, 15, 19] during periconception period, and 9 studies [7, 10–12, 14, 17, 18, 20] during all the pregnancy duration.

Women with type 1 diabetes showed a worse glycemic control than women with type 2 both in the preconception period and during pregnancy. 

Detailed data about metabolic control were reported in Table 2.

### 3.3. Obstetric, Fetal, and Neonatal Outcomes by Type of Diabetes

The pregnancy outcomes reported were preeclampsia [10–12, 16, 17, 19, 20], Caesarean section [10–13, 16–19], preterm delivery [10, 11, 16, 17, 19], miscarriages [15, 16, 20], stillbirth [8, 11, 13, 19], perinatal death [8, 10–14, 17–19], malformations [7, 8, 10–19], macrosomia [9, 10, 12, 14, 16, 18–20], and small for gestational age [12, 14, 17, 18, 20].

A comparison of data about obstetrical outcomes showed that preeclampsia and stillbirth rate is almost doubled in type 1 patients (9.7% and 2.8% versus 5.4% and 1.9%, resp.) while perinatal deaths and SGA importantly increased in type 2 diabetes (2.05% and 3.28% versus 3.36% and 5.7%, resp.). All the other obstetrical outcomes are comparable between diabetes types 1 and 2.

Detailed data about obstetrical outcomes were reported in Tables 3 and 4.

## 4. Discussion

The major difficulty in comparing the pregnancy outcomes in women with pregestational diabetes was to find articles with clear, suitable, and complete descriptions of maternal demographic characteristics and metabolic control before and during the pregnancy. 

In particular, a large portion of the published articles showed incomplete data in terms of preconception care rate and number of planned pregnancies. Many articles often considered only some of the possible pregnancy outcomes in women with pregestational diabetes.

Studies evaluating the potential predictors of adverse outcomes showed that a poor glycaemic control before and during pregnancy is a relevant factor influencing obstetrical, fetal, and neonatal outcomes [5, 21, 22]. 

Much evidence reported that maternal demographic characteristics (age, BMI, and duration of diabetes) similar to adequate preconception care could play a role in influencing and predicting pregnancy outcomes [23–27]. 

Preeclampsia is one of the most frequent pregnancy complications in the diabetic cohort of patients. The higher incidence of preeclampsia in analyzed studies was not so surprising even if the mean percentage of preeclampsia was 9.7% in women with type 1 and 5.4% in women with type 2. In fact, according to the well-accepted hypothesis explaining preeclampsia in diabetic women, it seems to be correlated with endothelial dysfunctions, insulin resistance, and poor glycemic control in early pregnancy [28, 29]. 

It is important to evaluate systemic comorbidities since there is evidence showing that nephropathy, usually more frequent in type 1 diabetes, is found to be an independent risk factor for preeclampsia onset. In fact, our data showed a twofold higher incidence of preeclampsia in type 1 diabetic women with respect to type 2. This evidence is confirmed by ACOG practice bulletin that observed a preeclampsia risk of 15–20% in pregnancies complicated by type 1 diabetes without nephropathy and approximately a risk of 50% in the presence of nephropathy [6].

Another obstetrical complication frequently reported in pregestational diabetic women is preterm delivery with comparable incidence between types 1 and 2 diabetes (33.6% in women with type 1 versus 32% in women with type 2). According to French multicentric survey (435 pregnancies in women with pregestational diabetes), diabetes is directly implied in preterm delivery risk particularly when first trimester HbA1c >8% occurs and a preexisting nephropathy is reported. Both conditions are responsible for increased risks of gestational hypertension and preeclampsia which are independently associated with preterm delivery [8]. So, pregestational diabetes could be directly responsible for ruptures of membranes and preterm delivery by amniotic fluid alteration and increased risk of ascending infections. Moreover, diabetes could be considered indirectly responsible for preterm delivery since pregestational diabetic pregnant women have an increased risk of preterm termination of pregnancy for obstetrical indication linked to impairment of fetal status up to intrauterine death: growth restriction, poor glycemic control, congenital malformation, macrosomia, nonreassuring fetal heart rate, acute polyhydramnios, and acute fatty liver of pregnancy [3, 10, 30]. 

In case of diabetic preterm delivery, newborns are more susceptible to perinatal complications than nondiabetic preterm ones since they present an increased risk of growth retardation, hypoglycemia, hypocalcaemia, polycythemia, hyperbilirubinemia, several types of malformations, hypertrophic cardiomyopathy, and asphyxia [3, 10, 30]. 

Our data showed that the mean prevalence of perinatal deaths was 2.05% in women with type 1 and 3.36% in women with type 2 while the mean prevalence of stillbirth was 2.8% in women with type 1 and 1.9% in women with type 2.

The high rate of perinatal deaths and stillbirth in offspring of diabetic women could have many explanations. 

First of all, some deaths are caused by major malformations, occurring in 6–12% of infants of women with pregestational diabetes [31].

Another important cause of fetal death is the metabolic acidosis and hypoxia due to the insufficient transplacental exchanges linked to an altered placental blood flow [25]. 

Rapid fetal growth or macrosomia, induced by the endogenous hyperinsulinemia, is related to perinatal deaths and stillbirth and could also be responsible for several intrapartum complications such as dystocia, birth trauma, respiratory distress syndrome, neonatal hypoglycemia, hyperbilirubinemia, and polycythemia [5].

We detected a mean prevalence of macrosomia in 22.3% of women with type 1 and 21.7% in women with type 2 diabetes. Murphy et al. demonstrated that the high levels of third trimester HbA1c and social disadvantage are the main risk factors for delivering an infant with macrosomia [20]. This concept is concordant with two studies that described a correlation between macrosomia and the postprandial third trimester glucose levels. 

More precisely, Combs et al. identified a target 1-hour postprandial glucose value of 7.3 mM (130 mg/dL), which may be the level that optimally reduces the incidence of macrosomia without increasing the incidence of SGA infants [32]. 

The risk of fetal hypoglycemia and subsequent implication in fetal growth represent an aspect which should not be underestimated since limitation in fetal glucose availability may compromise fetal nutrition. Recent studies show a correlation between low HbA1c levels and incidence of SGA since a lowered maternal glucose concentration in diabetic pregnants with retinopathy improves the ocular vascularization but limits the glucose supply to the fetus and may compromise fetal growth [33, 34]. 

The altered glycemia control could explain the increased intrauterine death rate in type 1 diabetic women, while the increased perinatal death rate in type 2 diabetic women seem not be related to glicemic control which is usually better in this cohort of patients. Some authors suggest that, in this cohort of patients, the high rate of perinatal deaths can be linked to high maternal BMI and advance age, frequently detected in this population [24–27, 35].

The higher percentage of CD reported in diabetic population with respect to general population can be explained at least in two ways. Firstly, the high percentage of obstetrical complications, both maternal and fetal, was responsible of high rate of urgent CD when nonreassuring fetal status occurs. Secondly, the known high rate of both intrauterine and perinatal complications up to death lead to Obstetricians to programs elective CD as soon as possible after estimation of fetal maturation and possible neonatal autonomy.

The worry of higher risk of fetal adverse events in diabetic women with respect to general population is linked to universally accepted evidence that embryos and fetuses developed in pregestational diabetic women are affected by high congenital malformations rate which is responsible for a major susceptibility in case of any fetal stress.

The high rate of congenital malformation explains also the high rate of miscarriages detected in this population of pregnants (13.4% in women with type 1 and 12.4% in women with type 2) [15, 36]. The mean prevalence of malformations was 5.3% in women with type 1 and 5.7% in women with type 2. The most common anomalies associated with preexisting diabetes involve cardiovascular system, central nervous system, face, and extremities [37, 38]. 

Actually, the pathophysiology of congenital malformations in fetus of women with preexisting diabetes is not completely understood, but many studies suggest a correlation with poor glycemic control, especially during the preconceptional period and first trimester of pregnancy [7, 8, 10–19]. 

In fact, the American College of Obstetricians and Gynecologist suggests that women with diabetes should have a preferable preconceptional and conceptional fasting serum level of glucose less than 95 mg/dL with a HbA1c no higher than 6% [6]. 

According to this recommendation, our data showed that a higher rate of fetal malformations occurred in women with first trimester HbA1c higher than 7%.

## 5. Conclusion

Available evidence concerning preconceptional diabetes (both types 1 and 2) highlights the importance to obtain optimal glycemic profile in preconception period and during pregnancy in order to decrease the risk of adverse maternal and fetal outcomes during pregnancy.

Moreover, all studies concluded that it may be impossible to identify thresholds of glycaemia that will make an absolute separation between normal and high risk pregnancies although it seems possible to identify thresholds that ensure better pregnancy outcomes.

It is important to maintain glucose levels as close to normal as possible. In particular a glycosylated hemoglobin concentration no higher than 6% before pregnancy and during the first trimester seems to decrease the risk of preterm delivery, miscarriages, malformations, stillbirth, and perinatal death. Instead, the glycemic levels during the third trimester seem to be correlated with birth weight, so a 1 h postprandial glucose value lower than 130 mg/dL prevents macrosomia without increasing the risk of a SGA.

Despite a milder glycemic disturbance, type 2 diabetes represents a serious condition in pregnant women. In fact, women with type 2 diabetes sometimes have worse pregnancy outcome than women with type 1 diabetes and this may be related to the fact that they are older and heavier.

In our opinion, both the preconceptional counseling and optimization of glycemic profile represent two fundamental steps to improve pregnancy outcomes in women with preexisting diabetes. All diabetic women must be informed about the teratogenesis associated with their metabolic disease. 

A systematic approach to family planning and the availability of preconception care for all women with diabetes who desire pregnancy could be an essential step for diabetic management program. 

## Figures and Tables

**Table 1:** Detailed data about maternal characteristics according to the pregnancy outcomes.

Author (year)	DM type	Age (years)	BMI (kg/m²)	Duration of diabetes (years)	Preconceptional care (%)	Planned pregnancy (%)	Nulliparous (%)	Duration of gestation (week)	Retinopathy (%)	Nephropathy (%)	Miscarriages (%)	Stillbirths (%)	Perinatal deaths (%)	Malformation (%)	Macrosomia (%)	SGA (%)
Suhonen et al. (2000) [7]	I = 488			14.5 ± 7.9*			50	37.3						10.3		
Boulot et al. (2003) [8]	I = 289				48.5				34.3	11.8			0.3	4.5		
	II = 146				24				2.7	4.8			2.1	3.4		
Endres et al. (2004) [9]	I + II = 74	31 ± 6*		12 ± 9*		53	39	37 ± 2*	16	11					28	
Evers et al. (2004) [10]	I = 323	30 ± 4*	25 ± 4*	13 ± 8*		84	52						2.8	8.8	52.5	
Jensen et al. (2004) [11]	I = 1215	28.8	22.9	12 (5–19)^‡^	58				7.3	6.4		2.1	3.1	5	8	
Clausen et al. (2005) [12]	I = 240	30 (27–33)^‡^	23 (21–26)^‡^	14 (6–19)^‡^			54	37.3 (36–38)^‡^	31	11			1.7	2.9	51	4
	II = 61	33.4 (31–38)^‡^	29.4 (27–35)^‡^	2 (1–5)^‡^			31	38 (37–39)^‡^	5	13			6.7	6.6	56	2
Huddle (2005) [13]	I = 172	30.5 ± 6.2*		6 ± 4.2*				36.6 ± 2*	17.4	7.6		3.9	3.9	0.6		
	II = 213	34.3 ± 5*		3.7 ± 3.1*				37 ± 1.9*	9.9	1.9		1.8	2.8	0.9		
Roland et al. (2005) [14]	I = 389	29.8 ± 5.5*	26.4 ± 4.4*		40.5						10		2.8	4.4	46.5	3.6
	II = 146	33.9 ± 5.2*	34.2 ± 7.3*		28.7						10		6.2	12.3	46.9	9.6
Galindo et al. (2006) [15]	I + II = 127	31.4 ± 5.4*	25.1 ± 5.9*		11.9		52.3				7.9			0.13		
De Valk et al. (2006) [16]	I = 48	34.0 ± 5*	31.7 ± 7.4*	3 (16)^‡^							13.6			5.1	21.7	
Hillman Gadea et al. (2006) [17]	I = 532	29.4 ± 4.7*	23.3 ± 3.1*	11.8 ± 7.1*	22.6			36.7 ± 1.7*					1.7	4.7	35.6	1
	II = 93	31.8 ± 5.5*	28.9 ± 6.5*	5.7 ± 6*	16.1			37.1 ± 1.6*					1.1	6.5	23.9	3.3
Gonzalez-Gonzalez et al. (2008) [18]	I = 257	29 (25–32)^‡^	24 (22–27)^‡^	10 (5–16)^‡^	22.5			37.4 ± 2.3*	12.3	5.1			1.97	6.22	20.6	2.9
	II = 147	34 (30–38)^‡^	33 (28–37)^‡^	6 (2–8)^‡^	9.5			38 ± 2.2*	0.7	1.4			2.75	6.8	13.8	2.2
Lapolla et al. (2008) [19]	I = 504	29.9 ± 4.8*	23.3 ± 3.4*	13.4 ± 8.3*	43.9				21.2	1.2	6.3	1.06	0.21	5.9	13.2	4.9
	II = 164	33.2 ± 4.8*	28.1 ± 3.4*	5.7 ± 5.9*	29.1				1.1	0.6	10.7	1.9	1.9	2	11.8	11.4
Murphy et al. (2011) [20]	I = 408	30 (21–38)^‡^	25 (21.4–31.8)^‡^	13 (4–27)^‡^	31.4	55.5	50.6	37.4 (34–38.6)^‡^	29.2	2.2	13.3				52.9	4.9
	II = 274	34 (26–40)^‡^	32.4 (24.1–42.4)^‡^	3 (1–9)^‡^	19.6	48.8	30.7	38.1 (35.6–39.3)^‡^	5.1	2.6	17				37.6	11.4

DM: diabetes mellitus.

BMI: body mass index.

SGA: small for gestational age.

*Results shown as mean ± standard deviation.

^‡^Results shown as median ± interquartile range.

**Table 2:** Detailed data about metabolic parameters according to the pregnancy outcomes.

Author (year)	Design	DM	Pre-HbA1c (%)	Booking HbA1c (%)	1st trimester HbA1c (%)	2nd trimester HbA1c (%)	3rd trimester HbA1c (%)	Miscarriages (%)	Stillbirths (%)	Perinatal deaths (%)	Malformation (%)	Macrosomia (%)	SGA (%)
Suhonen et al. (2000) [7]	Prospective study	I = 488			7.5 ± 1.4*						10.3		
Endres et al. (2004) [9]	Prospective study	I + II = 74		7.6 ± 2*			6.4 ± 1*					28	
Evers et al. (2004) [10]	Prospective study	I = 323			6.5 ± 1.0*	5.9 ± 0.9*	6.2 ± 1.1*			2.8	8.8	45.1	
Jensen et al. (2004) [11]	Prospective study	I = 1215	7.6		7.3 (6.6–8.2)^‡^	6.6 (6–8.3)^‡^	6.7 (6.2–7.4)^‡^		2.1	3.1	5		
Clausen et al. (2005) [12]	Retrospective study	I = 240			7 (6.5–7.8)^‡^	6.3 (5.8–6.8)^‡^	6.3 (5.9–6.7)^‡^			1.7	2.9	5	4
		II = 61			6.8 (6.1–7.7)^‡^	5.7 (5.2–6.2)^‡^	5.9 (5.5–6.2)^‡^			6.7	6.6	8	2
Huddle (2005) [13]	Retrospective study	I = 172		8.2 ± 2.0*			6.8 ± 1.0*		3.9	3.9	0.6		
		II = 213		7.8 ± 2.0*			6.5 ± 1.0*		1.8	2.8	0.9		
Roland et al. (2005) [14]	Prospective study	I = 389			7.35 ± 1.79*	6.49 ± 1.49*	6.51 ± 1.53*			2.8	4.4	46.5	3.6
		II = 146			7.22 ± 1.95*	6.09 ± 1.88*	6.58 ± 1.36*			6.2	12.3	46.9	9.6
Galindo et al. (2006) [15]	Prospective study	I + II = 127		6.5 ± 1.6*				7.9			7.4		
de Valk et al. (2006) [16]	Retrospective study	II = 48	6.9		6.4	6.0	6.2	13.6			5.1	3.3	
Hillman Gadea et al. (2006) [17]	Retrospective study	I = 532			7.2 ± 1.19*	6.3 ± 0.9*	6.2 ± 0.8*			1.7	4.7		1
		II = 93			6.4 ± 1.19*	5.8 ± 0.84*	5.8 ± 0.76*			1.1	6.5		3.3
Gonzalez-Gonzalez et al. (2008) [18]	Retrospective study	I = 257			7.1 ± 1.22*		6.0 ± 0.82*			1.97	6.22	20.6	2.9
		II = 147			6.6 ± 1.51*		5.5 ± 0.55*			2.75	6.80	13.8	2.2
Lapolla et al. (2008) [19]	Prospective study	I = 504		7.2 ± 1.6*					1.06	0.21	5.9	13.2	
		II = 164		6.4 ± 1.7*					1.9	1.9	2	11.8	
Murphy et al. (2011) [20]	Prospective study	I = 408	7.9 (6.5–11.1)^‡^		7.4 (6.1–9.6)^‡^	6.7 (5.7–8.2)^‡^	6.7 (5.6–8.1)^‡^	13.3					4.9
		II = 274	6.9 (5.7–10)^‡^		6.8 (5.6–8.5)^‡^	6 (5.2–7.1)^‡^	6.2 (5.2–7.3)^‡^	17					11.4

DM: diabetes mellitus.

SGA: small for gestational age.

HbA1c: haemoglobin glycated.

*Results shown as mean ± standard deviation.

^‡^Results shown as median ± interquartile range.

**Table 3:** Detailed data about obstetric, fetal, and neonatal outcomes by type of diabetes. Results shown as mean percentage (%).

Outcomes	Type 1 DM	Type 2 DM
Preeclampsia [10–12, 16, 17, 19, 20]	9.7	5.4
Preterm delivery [10, 11, 16, 17, 19]	33.6	32
Caesarian section [10–13, 16–19]	54.2	50.7
Miscarriages [15, 16, 20]	13.4	12.4
Stillbirth [8, 11, 13, 19]	2.8	1.9
Perinatal deaths [8, 10–14, 17–19]	2.05	3.36
Malformations [7, 8, 10–19]	5.3	5.7
Macrosomia [9, 10, 12, 14, 16, 18–20]	22.3	21.7
Small for gestational age [12, 14, 17, 18, 20]	3.28	5.7

DM: diabetes mellitus.

**Table 4:** Detailed data about obstetric outcomes according to pregnancy glycemic profile.

Author (year)	Design	DM (*n*)	1st trimester HbA1c (%)	2nd trimester HbA1c (%)	3rd trimester HbA1c (%)	Nephropathy (%)	Preeclampsia (%)	Caesarian section(%)	Preterm delivery (%)
Evers et al. (2004) [10]	Prospective study	I = 323	6.5 ± 1.0*	5.9 ± 0.9*	6.2 ± 1.1*		12.8	44.3	32.2
Jensen et al. (2004) [11]	Prospective study	I = 1215	7.3 (6.6–8.2)^‡^	6.6 (6–8.3)^‡^	6.7 (6.2–7.4)^‡^	6.4	18.1	55.9	41.7
Clausen et al. (2005) [12]	Retrospective study	I = 240	7 (6.5–7.8)^‡^	6.3 (5.8–6.8)^‡^	6.3 (5.9–6.7)^‡^	11	13	51	
		II = 61	6.8 (6.1–7.7)^‡^	5.7 (5.2–6.2)^‡^	5.9 (5.5–6.2)^‡^	13	7	36	
Huddle (2005) [13]	Retrospective study	I = 172			6.8 ± 1.0*	7.6		63	
		II = 213			6.5 ± 1.0*	1.9		61.5	
de Valk et al. (2006) [16]	Retrospective study	I = 48	6.4	6.0	6.2		8.9	42.9	21.4
Hillman Gadea et al. (2006) [17]	Retrospective study	I = 532	7.2 ± 1.19*	6.3 ± 0.9*	6.2 ± 0.8*		3.2	56	35.4
		II = 93	6.4 ± 1.19*	5.8 ± 0.84*	5.8 ± 0.76*		6.5	44.1	30.4
Gonzalez-Gonzalez et al. (2008) [18]	Retrospective study	I = 257	7.1 ± 1.22*		6.0 ± 0.82*	5.1		47.7	
		II = 147	6.6 ± 1.51*		5.5 ± 0.55*	1.4		42.8	
Lapolla et al. (2008) [19]	Prospective study	I = 504				1.2	4.3	73	37.5
		II = 164				0.6	3.1	69.3	33.6
Murphy et al. (2011) [20]	Prospective study	I = 408	7.4 (6.1–9.6)^‡^	6.7 (5.7–8.2)^‡^	6.7 (5.6–8.1)^‡^	2.2	7.8		
		II = 274	6.8 (5.6–8.5)^‡^	6 (5.2–7.1)^‡^	6.2 (5.2–7.3)^‡^	2.6	5.2		

DM: diabetes mellitus.

HbA1c: haemoglobin glycated.

*Results shown as mean ± standard deviation.

^‡^Results shown as median ± interquartile range.
